# Polygenic risk scoring to assess genetic overlap and protective factors influencing posttraumatic stress, depression, and chronic pain after motor vehicle collision trauma

**DOI:** 10.1038/s41398-021-01486-5

**Published:** 2021-06-29

**Authors:** Jarred J. Lobo, Samuel A. McLean, Andrew S. Tungate, David A. Peak, Robert A. Swor, Niels K. Rathlev, Phyllis L. Hendry, Sarah D. Linnstaedt

**Affiliations:** 1 Institute for Trauma Recovery, University of North Carolina, Chapel Hill, NC USA; 2 Department of Anesthesiology, University of North Carolina, Chapel Hill, NC USA; 3 Department of Emergency Medicine, University of North Carolina, Chapel Hill, NC USA; 4 Department of Emergency Medicine, Massachusetts General Hospital, Boston, USA; 5 Department of Emergency Medicine, Beaumont Hospital, Royal Oak, MI USA; 6 Department of Emergency Medicine, Baystate State Health System, Springfield, MA USA; 7 Department of Emergency Medicine, University of Florida College of Medicine, Jacksonville, FL USA

**Keywords:** Predictive markers, Personalized medicine, Depression, Clinical genetics, Molecular neuroscience

## Abstract

Posttraumatic stress (PTS), depressive symptoms (DS), and musculoskeletal pain (MSP) are common sequelae of trauma exposure. Although these adverse posttraumatic neuropsychiatric sequelae (APNS) are often studied separately, clinical comorbidity is high. In a cohort of European American motor vehicle collision (MVC) trauma survivors (*n* = 781), substantial PTS (≥33, IES-R), DS (≥26, CES-D), and MSP (≥4, 0–10 NRS) were identified via a 6-month survey. Genetic risk was estimated using polygenic risk scores (PRSs) calculated from the largest available GWAS datasets of PTSD, MDD, and back pain. We then assessed comorbidity and genetic risk influence for developing chronic PTS, DS, and MSP after MVC. Secondary analyses explored whether common social determinants of health ameliorate genetic vulnerability. We found that 6 months after MVC, nearly half 357/781 (46%) of the participants had substantial PTS, DS, and/or MSP, and overlap was common (PTS + MSP (23%), DS + MSP (18%), PTS + DS (12%)). Genetic risk predicted post-MVC outcomes. PTSD-PRSs predicted PTS and DS (*R*^2^ = 2.21% and 2.77%, *p*_adj_ < 0.01), MDD-PRSs predicted DS and MSP (*R*^2^ = 1.89%, *p*_adj_ < 0.01) and 0.79%, *p*_adj_ < 0.05), and back pain-PRS predicted MSP (*R*^2^ = 1.49%, *p*_adj_ < 0.01). Individuals in the highest quintile of PTSD-PRSs had 2.8 and 3.5 times the odds of developing PTS and DS vs. the lowest quintile (95% CI = 1.39–5.75 and 1.58–7.76). Among these high-risk individuals, those living in non-disadvantaged neighborhoods and with college education had 47% (*p* = 0.048) and 52% (*p* = 0.04) less risk of developing PTS, and those with high social support had 60% (*p* = 0.008) less risk of developing DS. Overall, genetic factors influence the risk of APNS after MVC, genetic risk of distinct APNS are overlapping, and specific social determinants greatly augment genetic risk of APNS development after MVC.

## Introduction

Adverse posttraumatic neuropsychiatric sequelae (APNS), such as posttraumatic stress (PTS), depressive symptoms (DS), and musculoskeletal pain (MSP), are common and morbid following traumatic experiences [1, 2]. One of the most common traumatic events in industrialized nations, motor vehicle collisions (MVCs), result in 50 million injuries worldwide and almost 4 million US emergency department (ED) visits each year [3, 4]. In the United States, ~90% of individuals presenting to the ED after MVC are discharged to home after evaluation and many subsequently develop APNS [5–7]. Although most studies of MVC trauma survivors have focused on the study of individual APNS, available literature indicates that clinical comorbidity between them is high [8–13]. Factors contributing to APNS comorbidity after MVC remain poorly understood.

Evidence from other settings suggests that shared genetic vulnerability factors may contribute to APNS comorbidity after MVC. For example, results from large genome-wide association studies (GWASs) estimate heritability for these outcomes to be 5–20% [14–16]. Polygenic risk scores (PRSs) provide a means of estimating aggregate genetic risk across many genes [17], such scores have been used to estimate genetic variation in individual APNS outcomes. For instance, Misganaw et al. [18] showed that posttraumatic stress disorder (PTSD)-PRS accounts for 4.68% of the variance in PTS development following combat trauma and Waszczuk et al. [19] showed that a re-experiencing PRS increases odds of PTS development by 1.21 in World Trade Center disaster emergency responders. In addition, Howard et al. [15] showed that genetic variants account for up to 3.2% of variance in major depressive disorder (MDD) diagnosis and van Reij et al. [20] showed that chronic widespread pain-PRS accounts for 6% of variance in chronic postsurgical pain outcomes. The extent to which genetic vulnerability is shared across these disorders is less well understood, because few studies have assessed multiple APNS simultaneously.

Social determinants of health are also known to influence vulnerability to APNS after MVC [21–24]. For example, social support can protect against adverse effects of stressful events [25, 26]. Earning a college degree may improve employment opportunities and raise income, which allows for access to higher-quality housing or healthcare resources, resulting in better health outcomes [27–31]. In addition, economically disadvantaged neighborhoods are disproportionately affected by environmental conditions that affect health [32–36]. The extent to which these social determinants mitigate genetic risk of post-MVC APNS is not understood. A better understanding of these relationships would allow clinicians to take into account an individual’s variability in genes, environment, and lifestyle when treating and preventing adverse posttraumatic outcomes [37].

In the current study, we used PRS methods to examine genetic vulnerability and shared genetic risk of PTS, DS, and MSP after MVC, using data from a longitudinal observational study of MVC trauma survivors presenting to the ED after MVC and discharged to home after evaluation. We hypothesized that PRS-calculated genetic vulnerability would partially account for clinical comorbidity in APNS development following MVC. In secondary analyses, we assessed the hypothesis that previously identified social determinants of health ameliorate genetic vulnerability.

## Methods

### Study design and population

The details of the MVC study have been reported previously [38]. In brief, European American individuals, ≥18 and ≤65 years of age, presenting to one of the eight EDs in four no-fault insurance states (where litigation related to persistent post-MVC pain is more restricted [39]) within 24 h of MVC, who did not have a fracture or other injury requiring hospital admission, were enrolled. Patients who were not alert and oriented were excluded, as were pregnant patients, prisoners, patients unable to read and understand English, or patients taking opioids above a total daily dose of 30 mg of oral morphine or equivalent. In addition, because genetic analyses are potentially biased by population stratification, enrollment was limited to self-reported non-Hispanic Whites (the most common ethnicity at study sites). Informed consent was obtained from all participants and Institutional Review Board approval was obtained at all study sites.

### Assessments

Demographic and crash characteristics (e.g., front- vs. rear-end collision, airbag deployment, driver vs. passenger) were assessed via patient interview. Information regarding the time of the MVC and the time of ED presentation were abstracted from the medical record.

MVC-related PTS symptoms were assessed 6 months following MVC using the Impact of Event Scale: Revised [40]. This 22-item questionnaire includes avoidance, intrusion, and hyperarousal subscales. Scores range from 0 to 88; a validated cutoff of 33 was used to define individuals with substantial PTS vs. individuals with low PTS scores [41].

MVC-related DS was assessed 6 months following MVC using the Center for Epidemiological Studies Depression (CES-D) Scale, a 20-item self-report questionnaire designed to assess symptoms of depression [42]. CES-D scores range from 0 to 60, with scores of 26 or more indicating substantial DS [43].

MVC-related overall pain intensity in the past week was assessed via web-based questionnaire or telephone interview 6 months following MVC, using a verbal 0–10 numeric rating scale (NRS). Verbal scores have advantages in acute care settings and verbally administered NRSs have been validated as a substitute for visual analog scales in acute pain measurement in the ED [44]. If participants reported pain, they were also asked whether the pain was related to the MVC; only MVC-related pain (>99% of all pain reported) was included in the present analyses. NRS scores ≥ 4 were used to define individuals with moderate–severe pain [45].

A socioeconomic position (SEP) index, used to assess neighborhood socioeconomic status (SES), was generated for each participant as previously described [33]. In brief, participant addresses at the time of the ED visit and census tract data from the 2010 American Community Survey were used to generate SEP indexes based on five measures of deprivation: percent unemployed, percent below the US poverty line, percent with high school education or less, percent expensive homes (owner-occupied homes worth ≥ $300,000) in the neighborhood, and median household income [46]. SEP index scores were split into quartiles; individuals with scores in the first and second quartiles were said to live in “disadvantaged neighborhoods” and individuals with scores in the third and fourth quartiles were said to live in “non-disadvantaged neighborhoods” (Supplementary Fig. S1).

Perceived social support was assessed using the “significant other” subscale of the multidimensional scale of perceived social support [47]. Four statements such as, e.g., “there is a special person who is around when you are in need” were presented to the participants and they responded with their level of agreement with the statement on a scale of 1–7, with the higher the number corresponding to the strongest agreement with the statement. Scores from the four statements were averaged and scores ≤ 5 were defined as “low social support” and scores > 5 as “high social support,” as described previously [48] (Supplementary Fig. S1).

Educational attainment was obtained via patient self-report during the research interview. Patients were asked to select their educational attainment from seven categories based on their highest grade obtained. Patients with “some college or less” (the median) were compared to patients with a “college education.” (Supplementary Fig. S1).

MVC severity was assessed via patient self-report. Patients were asked “which of the following phrases best describes the extent of damage, overall, to the vehicle you were traveling in?” Patients selected from “minor,” “moderate,” or “severe” accident severity.

### DNA collection from MVC study participants and SNP genotyping

Blood samples were collected from participants into PAXgene DNA tubes at the time of study enrollment. DNA was extracted from blood using PAXgene blood DNA kits (QIAGEN, Inc., Germantown, MD). Genotyping of purified DNA was performed using the Axiom Precision Medicine Research Array (PMRA, 902,560 genetic markers; ThermoFisher Scientific) at McGill University, Montreal, Canada. Three samples with known genotype (1000 Genomes), 1 blank, and 1 repeat sample were included in each genotyping batch (96 samples), to ensure genotypic accuracy and reliability.

Genetic data cleaning was performed using established pipelines [49]. In brief, all genotyped samples were assessed for identity and quality via call rate, chromosomal anomalies, cryptic relatedness (using kinship coefficients, R package SNPRelate), autosomal heterozygosity, gender mismatch, and genetic ancestry. Batch effects were assessed by comparing missing call rate and *χ*^2^-test for allelic frequency. The median sample call rate was 99.9%. Samples were excluded if there was a discrepancy between annotated and genetic sex, chromosomal abnormalities, and kinship coefficients > 0.354 without known relation or sample duplication. Single nucleotide polymorphism (SNP) quality checks included assessments of missing call rate, duplicate discordance, and Mendelian errors. SNPs were filtered for Hardy–Weinberg equilibrium if *p* < 0.00001 and minor allele frequency (MAF) if MAF < 0.01. A total of 884,652 SNPs were retained (out of 902,560 on the PMRA) and were used for subsequent genetic imputation. Cleaned genotypes were then imputed to the 1000 Genomes Project phase 3 reference panel [50], using the software packages SHAPEIT2 [51] for pre-phasing and IMPUTE2 [52] for imputation.

### Generation of PRSs

GWAS summary statistics from the largest available GWAS analyses of PTSD (*n* = 23,212 cases and *n* = 151,447 controls) [53], depression (*n* = 246,363 cases and *n* = 561,190 controls) [54], and back pain (*n* = 119,384 cases and *n* = 334,478 controls) [55], were used to generate PRSs in the MVC trauma cohort. PRS for PTS, DS, and MSP were generated using each PTSD, depression, and back-pain GWAS. Genetic overlap was examined between outcomes by using the summary statistics from a GWAS of one trait to generate a PRS that predicts genetic vulnerability to another trait [56]. The back-pain GWAS was used to generate a PRS for MSP, because it is the largest study to date, which can represent MSP (the back, neck/shoulder, hip, and knee). Only summary statistics data from White individuals were used to match ancestry of discovery and target samples [57].

PRS were calculated, evaluated, and plotted using PRSice v2.3.3 [58]. Briefly, the software generates a PRS by summing all trait-associated alleles in a target sample, weighted by the effect size of each allele in the base GWAS. To select the optimal set of trait-associated alleles, linkage disequilibrium (LD) clumping and *p*-value thresholding were used. Imputed base SNPs were filtered with information scores < 0.9 and MAF < 0.01. In addition to the genetic data cleaning, imputed target SNPs were filtered with information scores < 0.9. SNPs in LD were grouped, to not give extra weight to a single marker. The most representative SNP with the smallest *p*-value was chosen within a given 250 kb window with *r*^2^ > 0.1. PRS were generated at incrementally increasing *p*-value thresholds in the base GWAS and the optimal threshold, explaining the most variance in the target sample, was selected (Supplementary Fig. S2). Previous research indicates that PRSice is well-powered to detect the cumulative effect of SNPs in target sample sizes of at least 100 subjects and base sample sizes of at least 50,000 subjects [58] (Supplementary Fig. 2). In this study, we calculated PRS in our MVC cohort (*N* = 781) using summary statistics from three large GWAS (*N* = 174,657 to *N* = 807,553).

### Statistical analyses

Sociodemographic characteristics were summarized using standard descriptive statistics. A Venn diagram was created to visualize the overlap in PTS, DS, and MSP outcomes 6 months following MVC trauma. Each circle represents a different outcome and overlapping circles indicate participants who had two or more outcomes.

To maintain consistency with the GWAS from which the PRS were derived, we used a similar case–control set-up and logistic regressions to test our PRS models. Assuming a population prevalence of 10% after trauma exposure, odds ratio (OR) of 2, and calculated *ρ*^2^ of 0.12, our study met the minimum sample size of *N* = 205, which is required to estimate the main effect of PRS on PTS, DS, or MSP using a logistic regression [59, 60]. Here, *ρ*^2^ was calculated using a multiple linear regression model where PRS was the outcome and neighborhood SES, social support, educational attainment, age, sex, site, and top ten principal components were predictors. *R*^2^ was calculated to be 0.08 in the model with PRS for PTS and 0.12 for the model with PRS for DS. As it is more stringent, 0.12 was used as *ρ*^2^ in the sample size calculation. Both the OR per quintile of PRS and Nagelkerke’s pseudo-*R*^2^ were calculated to estimate the effect of each PRS. Nagelkerke’s pseudo-*R*^2^ of the PRS was calculated as the difference between the Nagelkerke’s pseudo-*R*^2^ of the full model that contains PRS along with covariates (sex, age, site, and top ten principal components) and the Nagelkerke’s pseudo-*R*^2^ of the null model that contains only covariates. We adjusted for multiple testing using a false discovery rate of 5%, as we had three independent but related outcomes of PTS, DS, and MSP following MVC. Accident severity was not a significant adjuster in patients with available data (Supplementary Table S1), which is consistent with other PRS studies on PTSD [19].

The PRSs derived from the PTSD GWAS best explained the variance in PTS and DS. Therefore, these PRSs were used for secondary analyses to examine how neighborhood SES, perceived social support, and college education protect against genetic vulnerability to PTS and/or DS. PRS were normally distributed and standardized using R (mean = 0, SD = 1) (Supplementary Fig. S3). Logistic regression modeling was used to assess the main effects of both the PRSs and each protective factor, adjusting for age, sex, study site, and top ten genetic principal components, with PTS and DS as the dependent variables.

To investigate the effect of each protective factor within subgroups, PRS quintiles for PTS and DS were used to create three risk groups: low (quintile 1), mid (quintiles 2–4), and high (quintile 5), as previous studies show that the greatest protection or risk is at the extremes of polygenic risk [61]. Neighborhood SES, perceived social support, and college education were evaluated within each polygenic risk group as protective factors that offset genetic risk to PTS and/or DS using equality of proportions with Yates’ continuity correction (*prop.test* in R). The required sample size to achieve an 80% power is *N* = 156 for a significance level *α* = 0.05, approximately equal sample size in each group, and the probability of group 1 being 10% and group 2 being 25% (*power.prop.test* in R). Each test had at least 156 participants.

## Results

### Participants

Baseline characteristics of participants included in the current study (*n* = 781) are shown in Table 1. Most participants were women <40 years of age with at least some college education and were overweight (body mass index > 25).

**Table 1:** Baseline characteristics of target cohort (*n* = 781).

Characteristic	
Age, years, mean (SD)	36 (13)
Women, *n* (%)	489 (63)
Education, *n* (%)	
HS or less	222 (28)
Some college	252 (32)
College	201 (26)
Post-college	105 (13)
Collision characteristics, *n* (%)	
Driver	676 (87)
Airbag deployed	219 (29)
Front end	359 (46)
Severe vehicle damage	408 (54)
BMI, mean (SD)	28 (6)
Current smoker, *n* (%)	200 (26)
Distress^a^ in ED, mean (SD)	19 (10)
Pain severity^b^ in ED, mean (SD)	5.5 (2.4)

*BMI* body mass index, *ED* emergency department, *MVC* motor vehicle collision trauma, *SEP* socioeconomic position.

^a^Distress was measured with the peritraumatic distress inventory (scale of 0–52).

^b^Pain severity was measured using the numeric rating scale (0–10 NRS).

### Adverse neuropsychiatric outcomes and clinical comorbidity between these outcomes were common 6 months following MVC

**Fig. 1:**
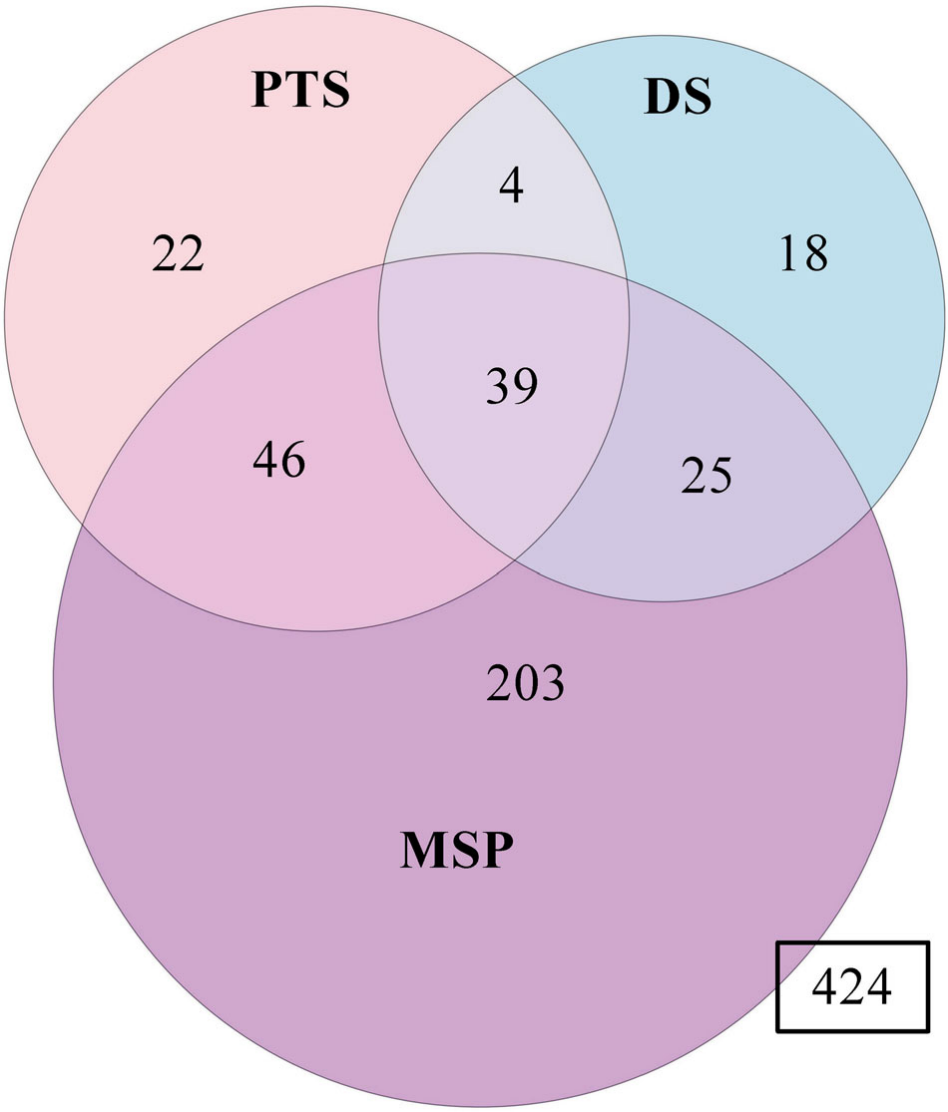
Overlap in posttraumatic stress (PTS), depressive symptoms (DS), and musculoskeletal pain (MSP) outcomes 6 months following motor vehicle collision trauma in White individuals (*n* = 781). Clinical overlap between common adverse posttraumatic neuropsychiatric outcomes is represented here via Venn Diagram. In total, 357 (46%) participants experienced at least one outcome and 424 participants reported full recovery (i.e., no PTS, DS, or MSP). Of those individuals experiencing at least one outcome, 32% (*n* = 114/357) reported two or more outcomes and 11% (*n* = 39/357) reported all three outcomes. Further details/summaries are presented in the main body of the manuscript.

Although the majority of individuals had recovered 6 months following MVC (*n* = 424, 54.3%), a substantial subset reported adverse neuropsychiatric symptoms (Fig. 1). Moderate-to-severe MSP was the most prevalent outcome (*n* = 313, 40.0%), followed by PTS (*n* = 111, 14.2%) and DS (*n* = 86, 11.0%). Clinical overlap in these outcomes was common, with 32% (*n* = 114/357) of individuals reporting two or more adverse neuropsychiatric symptoms. Of this clinical overlap, 23% (*n* = 85/357) reported both PTS and MSP, 18% (*n* = 64/357) reported both MSP and DS, and 12% (*n* = 43/357) reported PTS and DS. Eleven percent (*n* = 39/357) reported all three outcomes.

### PRSs generated using summary statistics from large genetic association studies of PTSD, MDD, and back pain predicted PTS, DS, and MSP outcomes 6 months following MVC

PRSs for 6-month PTS, DS, and MSP outcomes were generated using summary statistics from the three largest GWASs of PTSD, MDD, and back pain to date. The PRSs generated from the PTSD GWAS explained 2.21% of the variance in 6-month PTS (*p*_raw_ = 1.9 × 10^−3^, *p*_adj_ = 5.6 × 10^−3^) and 2.77% of the variance in 6-month DS (*p*_raw_ = 1.0 × 10^−3^, *p*_adj_ = 3.1 × 10^−3^) (Table 2). The PRSs generated from the MDD GWAS explained 1.89% (*p*_raw_ = 6.3 × 10^−3^, *p*_adj_ = 9.5 × 10^−3^) variance in 6-month DS and 0.79% variance for the MSP outcome (*p*_raw_ = 0.03, *p*_adj_ = 0.045). Finally, the back-pain GWAS explained 1.49% variance in 6-month MSP (*p*_raw_ = 2.9 × 10^−3^, *p*_adj_ = 8.7 × 10^−3^) but did not explain variance for PTS or DS.

**Table 2:** Variance (Nagelkerke’s pseudo-*R*^2^) explained by PRS in predicting PTS, DS, and MSP 6 months following MVC trauma in a cohort of European American individuals.

	Symptoms 6 months following MVC		
GWAS	PTS	DS	MSP
PTSD	2.21%^**^	2.77%^**^	0.32%
MDD	0.70%^***^	1.89%^**^	0.79%^*^
Back pain	0.15%	0.94%^***^	1.49%^**^

PRSs were generated from the three largest GWASs of PTSD, MDD, and back pain available to date. In the MVC cohort, PTS was defined 6 months following MVC using a validated cutoff of 33 on the IES-R. DS was defined using a validated cutoff of 26 on the CES-D Scale, indicating substantial depressive symptoms. MSP was defined as moderate-to-severe MVC-related overall pain intensity in the week prior to the 6-month follow-up timepoint using a cutoff of 4 on a 0–10 numeric rating scale. Details of the GWAS studies used to generate PRSs are described in the “Methods” section. The *R*^2^ reported is the *R*^2^ of the full model (PRS and age, sex, and site covariates) minus the *R*^2^ of the null model (covariates only).

*CES-D* Center for Epidemiological Studies Depression, *DS* depressive symptoms, *GWASs* genome-wide association studies, *IES-R* Impact of Event Scale: Revised, *MSP* musculoskeletal pain, *MVC* motor vehicle collision, *PRS* polygenic risk scores, *PTS* posttraumatic stress.

*p < 0.1.

**p < 0.05.

***p < 0.01. (FDR adjusted).

**Fig. 2:**
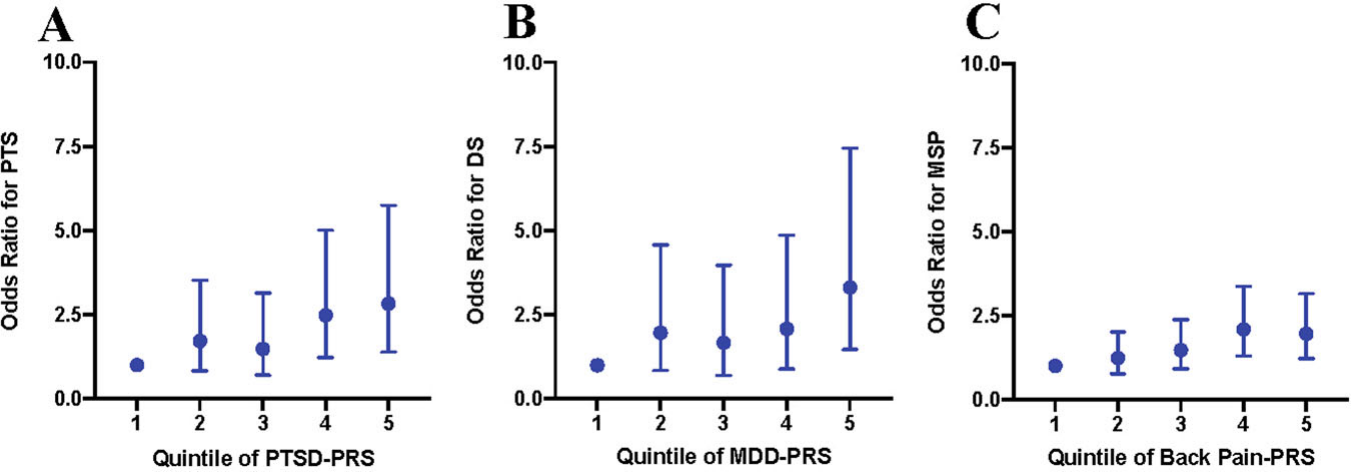
Odds ratios for each quintile of the polygenic risk scores for PTS, DS, and MSP as derived using the respective PTSD, depression, and back-pain GWAS summary statistics. The first quintile is used as a reference. For every other quintile, the odds ratio relative to the first quintile is displayed for **A** posttraumatic stress (PTS), **B** depressive symptoms (DS), and **C** musculoskeletal pain (MSP). For PTS, individuals in the fifth quintile have 2.8 times higher odds of developing PTS than individuals in the first quintile. The error bars indicate 95% confidence intervals around the odds ratios.

Using PRSs for PTS, DS, and MSP generated from GWAS summary statistics of the matching disorder, participants were stratified into risk quintiles for each 6-month outcome following MVC (Fig. 2). The highest quintile of risk substantially increased the odds of developing PTS (OR = 2.83, 95% confidence interval (95% CI) = 1.39–5.75), DS (OR = 3.31, 95% CI = 1.47–7.45), and MSP (OR = 1.96, 95% CI = 1.22–3.16) when compared to the lowest quintile. Genetic overlap was also identified between PTS and DS: being in the highest vs. lowest quintile of PTSD-PRS increased the odds of developing DS after MVC (OR = 3.50, 95% CI = 1.58–7.76) and being in the highest vs. lowest quintile of MDD-PRS increased the odds of developing PTS after MVC (OR = 2.20, 95% CI = 1.15–4.21).

### Secondary analyses using the PTSD GWAS PRS and the PTS and DS outcomes to establish whether social determinants of health protect against genetic risk of these outcomes

In secondary analyses, we assessed whether three social determinants of health [23, 24] (living in a non-disadvantaged neighborhood, high social support, and college education) could offset genetic vulnerability (assessed using PTSD-PRS, because these risk scores best explained variance in PTS and DS) to developing APNS after MVC, using logistic regression models adjusted for age, sex, study site, and top ten genetic principal components (Supplementary Tables S2 and S3). PTSD-PRS for PTS and DS were not associated with the three environmental factors (Supplementary Table S4). In the model evaluating the influence of genetic risk and social support on the development of substantial chronic DS 6 months after MVC, as expected, genetic risk increased the odds of 6-month DS (adjusted OR (aOR) = 1.54 per 1 SD increase in polygenic risk, 95% CI = 1.20–2.00, *p*_raw_ = 0.001, *p*_adj_ = 0.001) and high social support decreased the odds of 6-month DS (aOR = 0.38, 95% CI = 0.23–0.62, *p*_raw_ = 8.72 × 10^−5^, *p*_adj_ = 2.62 × 10^−4^). Similarly, in the other models, genetic risk increased the odds of 6-month PTS or DS and the three social determinants were associated with reduced odds of 6-month PTS and/or DS.

**Fig. 3:**
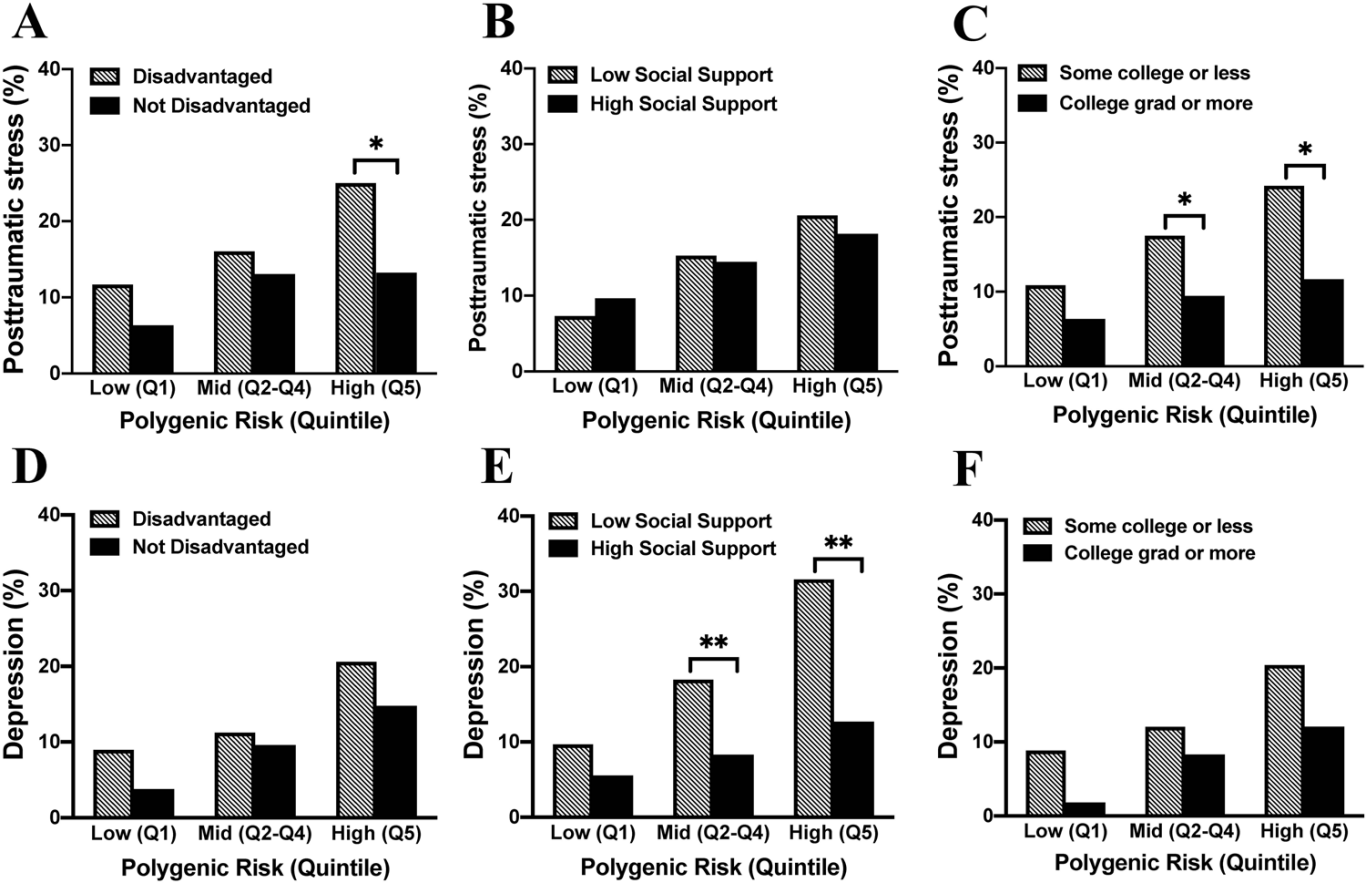
Prevalence of posttraumatic stress (PTS) and depressive symptoms (DS) 6 months following motor vehicle collision (MVC) trauma based on PTSD GWAS polygenic risk scores and the effect that three social factors have on this genetic vulnerability to PTS and DS. Prevalence of PTS (**A**–**C**) and DS (**D**–**F**) were graphed for each polygenic risk group (defined by quintiles as presented in Supplementary Fig. S4, with the second, third, and fourth quintiles grouped in the “Mid” risk category). **A**, **D** Influence of living in a disadvantaged neighborhood (striped bars) vs. a non-disadvantaged neighborhood (black bars) on genetic risk for PTS and DS. **B**, **E** Influence of social support on genetic risk for PTS and DS (striped bars represent low social support and black bars represent high social support). **C**, **F** Influence of education level on genetic risk for PTS and DS (striped bars represent some college or less and black bars represent college education or higher). Significance of equality of proportions models within each polygenic risk category are indicated above relevant bars; *p < 0.05, **p < 0.01.

We then assessed whether social determinants of health mitigated genetic risk of developing chronic PTS and/or DS after MVC. To best evaluate this, we focused on the highest vs. lowest genetic risk quintiles, as greatest genetic risk/protection is at the extremes of risk [61] (Supplementary Fig. S3). Individuals in the highest vs. lowest quintiles of genetic risk had increased odds of developing PTS (OR = 2.83, 95% CI = 1.39–5.75) and DS (OR = 3.50, 95% CI = 1.58–7.76) (Supplementary Fig. S4). Among individuals with the highest genetic risk, a significant difference was observed between living in a non-disadvantaged neighborhood and the development of chronic PTS after MVC (11/83(13.3%) vs. 18/72(25.0%), *p* = 0.048). Individuals living in a non-disadvantaged neighborhood had 47% less risk of developing PTS compared to individuals living in a disadvantaged neighborhood. In contrast, no difference was observed in the incidence of DS according to neighborhood SES (Fig. 3A, D).

We next assessed whether a high level of social support mitigated genetic risk for PTS and DS. For PTS, no statistically significant difference was observed between low and high social support and genetic risk (Fig. 3B). For DS, differences were observed between high and low levels of social support and moderate or high levels of genetic risk (moderate genetic risk: (30/362 (8.3%) vs. 19/104 (18.3%), *p* = 0.003), high genetic risk: (15/118 (12.7%) vs. 12/38 (31.6%), *p* = 0.008, Fig. 3E). Among individuals with the highest genetic risk, those with high social support had 60% less risk of developing DS compared to individuals with low social support.

Finally, we assessed whether college education mitigated genetic risk for PTS and DS. For PTS, individuals with middle and high genetic risk and college education had a lower risk for PTS (middle genetic risk: high educational attainment 9.4% (*n* = 17/180) vs. those with some college or less, 17.5% (*n* = 50/285, *p* = 0.01); high genetic risk: college education, 11.7% (*n* = (7/60) vs. some college or less 24.2% (*n* = 23/95, *p* = 0.04)). Among individuals with the highest genetic risk, those with college education had 52% less risk of developing PTS compared to individuals with some college or less. No statistically significant difference due to college education was observed for the low-risk group. We found a statistically significant difference in prevalence of PTS but not DS when comparing individuals with college education vs. those with some college or less in middle and high genetic risk groups (Fig. 3C, F).

## Discussion

The findings of this study demonstrate that genetic risk, assessed using polygenic risk scoring, can substantially influence an individual’s likelihood of developing PTS, DS, and/or MSP after a MVC trauma. Individuals experiencing MVC with the highest genetic risk had up to 3.5 times the odds of developing APNS vs. individuals with the lowest risk. Further, we showed that genetic risk of developing APNS after MVC is overlapping, demonstrating that vulnerability to comorbid APNS outcomes after MVC is partly genetic in origin. Finally, we found that specific modifiable environmental risk factors interact with genetic vulnerability, possibly decreasing the likelihood of an individual with genetic vulnerability, who is involved in an MVC developing one or more APNS. These findings have several important clinical and etiological implications.

First, our finding that overlapping vulnerability to comorbid outcomes is partly genetic in origin provides further evidence that biologic factors/pathways influencing APNS pathogenesis may be shared. This is important, for several reasons. First, it provides further evidence that research advances regarding biological mechanisms mediating one APNS outcome may provide useful ideas regarding potential factors involved in other outcomes. Similarly, it suggests that studies examining biological mechanisms of APNS development after MVC (or other traumatic stressors) should collect outcome data on multiple APNS outcomes. In addition, these data provide further support for the concept that new medications targeting one of the APNS should be assessed for potential utility in others, in case they target mechanisms or biological pathways common to multiple APNS.

Second, clinical outcome data from our cohort demonstrate that PTS, DS, and MSP are common among patients seen in the ED after MVC and discharged to home, and that there is substantial clinical comorbidity between these APNS. Indeed, one-third of the men and women meeting criteria for one of these outcomes also meet criteria for at least one other. This high clinical comorbidity between multiple APNS has important implications for the clinical diagnosis and treatment of trauma survivors [62, 63]. This is because treating a single APNS in isolation is unlikely to serve patients best. Progress in the creation and testing of care pathways for a specific APNS after MVC, which factor into other APNS [64], are promising therapeutic avenues that align with these data regarding the patient experience. Therefore, identification of the shared (and the unique) underlying biological processes that influences vulnerability to comorbid (and singly occurring) outcomes of trauma is likely to lead to improved risk assessment and therapeutics for trauma survivors.

Third, although in the world of research professionals it has been well-demonstrated that biopsychosocial factors, distinct from, e.g., compensation-seeking, are the dominant driver of APNS development after MVC [21, 65–68], unfortunately in the lay public this remains a common view. Our findings demonstrate that genetic risk substantially increases the risk of APNS development after MVC, as it does for other medical conditions. Further, genetic and environmental factors affect outcomes, just as they do for other medical conditions. These data hopefully are a useful addition to a body of evidence for patients and other stakeholders that APNS after MVC are maladies just like any other, with complex biopsychosocial causes, and not merely cultural phenomena/attention [69] or compensation-seeking.

Although we were able to show genetic overlap between comorbid APNS, it is important to note that, similar to other studies [70], the amount of genetic overlap identified in our study does not fully explain the clinical comorbidity observed between PTS, DS, and MSP. Clinical overlap is also likely due to shared demographic and environmental factors such as age, sex, SES [71], social support [72], and education level [71]. However, for the portion of the variability that is explained by genetics, the current state of the science is a limiting factor. In this study, PRSs only capture a small proportion (up to 2.77% variance explained) of genetic influence on PTS, DS, and MSP. Data from twin studies have estimated the heritability of these disorders to be as high as 50% [73–77]. (However, GWAS analyses assessing the association between common gene variants and APNS estimate heritability to be between 5 and 20% [14–16].) In our study, a PTSD-PRS accounted for 2.21% of the variance in PTS, which is in line with the PTSD-PRS that accounted for 4.68% of the variance in PTSD in Misganaw et al. [57]. Our MDD-PRS accounted for 1.89% of the variance in DS, which is expected given the significantly smaller sample size when compared to the MDD-PRS in Howard et al. [15], which accounted for 3.2% of the variance in MDD diagnosis.

The GWAS studies from which our PRS scores were derived are small compared to sample sizes for maximal statistical power [78, 79]. As GWAS studies are highly dependent on sample size [80], as available GWAS studies for APNS outcomes increase, measures of genetic risk (PRSs) will become more accurate. In addition, as GWAS are developed for common traumatic stressors that match the environmental exposure of interest (e.g., MVC) and the specific APNS, this will also improve scoring accuracy. For example, the largest GWAS for pain to date is focused on back pain, which can have vastly different etiology vs. post-MVC pain[81], and the largest GWAS for depression captures a heterogeneous set of phenotypes that may differ from posttraumatic DS [82]. The etiological difference between MDD and posttraumatic DS may explain why PTSD-PRS explained more variance in DS than MDD-PRS. Posttraumatic DS may be more similar etiologically to PTSD than MDD.

Our finding that modifiable environmental factors interact with genetic risk is consistent with results of other studies examining these outcomes in other settings. Choi et al. [61] found that physical activity offsets genetic risk for depression, even in those individuals with the highest genetic risk. Tamman et al. [83] found that attachment style, another social environmental factor, interacts with genetic risk to influence PTSD. Another study by Choi et al. [84] found that unit cohesion (an index of perceived support and morale) was protective against depression, even among soldiers at highest genetic risk. Their observation is most similar to our finding that social support offsets genetic risk to DS after MVC among individuals with intermediate and high genetic risk, and supports social support as a modifiable environmental factor across different traumatic events and settings.

Our findings that living in a non-disadvantaged neighborhood mitigates genetic risk for PTS, but not DS, are consistent with previous reports indicating an association between SES and PTS [85] but not between SES and depression [34, 86]. Conversely, we found that high social support mitigated risk for DS but not PTS. This is consistent with previous reports indicating that social support buffers against depression in individuals experiencing high levels of life stress [87] and social support improves but does not prevent PTSD symptoms [88]. Finally, we found that higher educational attainment mitigated risk for PTS and not DS. (Due to the poor prediction of MSP with the PTSD-PRS, we did not assess for mitigation with this outcome of MVC.) This work provides a number of specific demonstrations regarding how biopsychosocial factors interact to shape post-MVC ANPS outcomes. The findings also provide evidence of health disparities, as among individuals with high genetic risk, living in a non-disadvantaged neighborhood and having high social support and educational attainment reduces risk of APNS after MVC. Neighborhood, social support, and educational attainment are modifiable environmental factors that can be the target of policy interventions.

Strengths of the present study include a prospective study design, a single type of trauma exposure, inclusion of both men and women, and additional assessment of psychosocial factors. The focus of the study on White individuals was both a strength and a weakness, in that it increased genetic homogeneity to evaluate study hypotheses with increased power, yet also prevents generalizability of study findings to other groups. Further studies of mixed ancestry groups/diverse groups are necessary. Second, because of our limited sample size, we did not stratify by sex. This might be valuable, as previous studies have shown that APNS such as PTS are more heritable in women than in men [89]. Third, we did not adjust for medication use in our analyses. Use of, e.g., opioids or SSRIs (selective serotonin reuptake inhibitors) could influence the underlying biology that then influences APNS outcome rates. We were also unable to adjust for lifetime number of traumatic events, which can also influence APNS development. Fourth, as noted above, genetic risks scores were derived from GWAS studies that were relatively small and not MVC/trauma-specific. Future studies should derive PRS from larger and more specific GWAS studies. Fifth, for the multiple PRS derived from a single GWAS (e.g., PTSD-PRS) to predict multiple outcomes of MVC (e.g., PTS, DS), the set of SNPs included in each PRS were not identical. However, based on how the PRS are calculated, the most predictive SNPs were overlapping and indicate shared genetics, whereas some less predictive SNPs differed. Sixth, we did not adjust for multiple hypothesis testing in secondary, exploratory analyses. Therefore, these analyses should be repeated in additional/larger cohorts. Finally, the generalizability of our findings to development of APNS following other traumatic experiences including combat or sexual assault is not clear. Future research should incorporate survivors of other types of trauma.

In conclusion, genetic risk can substantially influence an individual’s likelihood of developing PTS, DS, and/or MSP after a MVC trauma. Genetic risk of developing APNS after MVC is overlapping, demonstrating that vulnerability to comorbid APNS outcomes after MVC is partly genetic in origin and specific modifiable environmental risk factors interact with genetic vulnerability, greatly reducing the likelihood of an individual with genetic vulnerability, who is involved in an MVC, develops one or more APNS. Further studies are needed to understand how specific and aggregate genetic risks influence APNS development after MVC, how PRSs can increase accuracy in assessing individual risk for APNS via clinical risk prediction tools [90], and how biopsychosocial interactions with genetic risk determine recovery to health vs. chronic morbidity and suffering after MVC.

## Supplementary information

Supplementary Info
