# Supplementary material for: Polygenic risk scoring to assess genetic overlap and protective factors influencing posttraumatic stress, depression, and chronic pain after motor vehicle collision trauma
Source: Transl Psychiatry. 2021 Jun 29;11:359. doi: 10.1038/s41398-021-01486-5 (PMC8257703; doi:10.1038/s41398-021-01486-5)
Supplement: Supplementary file 1 — Supplementary Info [file 41398_2021_1486_MOESM1_ESM.docx]

**Polygenic risk scoring to assess genetic overlap and protective factors influencing posttraumatic stress, depression, and chronic pain after motor vehicle collision trauma**

Supplementary Materials

**Supplementary Figure S1.** Distribution of (A) neighborhood socioeconomic position (SEP) index scores, (B) multidimensional scale of perceived social support (MSPSS) on the “significant other” subscale, and (C) educational attainment (highest grade completed). Disadvantaged neighborhood, low social support, and some college or less are represented by striped bars. Non-disadvantaged neighborhood, high social support, and college education or higher are represented by black bars.

**B**
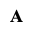


**A**

**C**
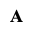


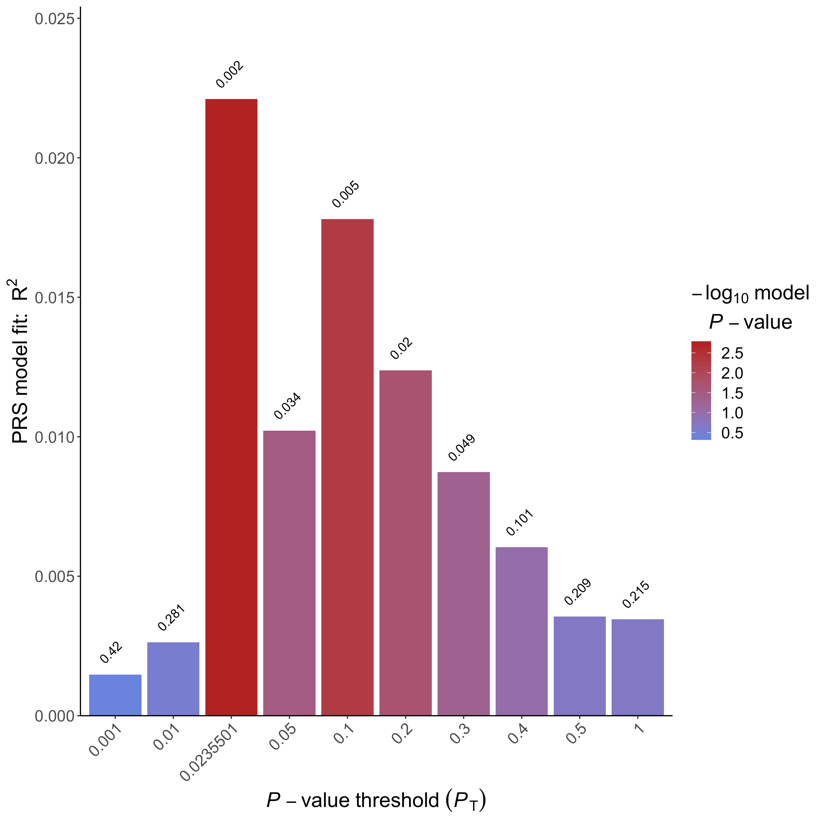
**Supplementary Figure S2.** Assessing the explanatory power of (A) posttraumatic stress, (B) depressive symptoms, and (C) musculoskeletal pain polygenic risk score models using p-value thresholding to select the optimal threshold after LD clumping. Models were adjusted for age, sex, site, and top 10 principal components. PRS were derived using the respective PTSD, depression, and back pain GWAS summary statistics.

**A**

**B**

**C**


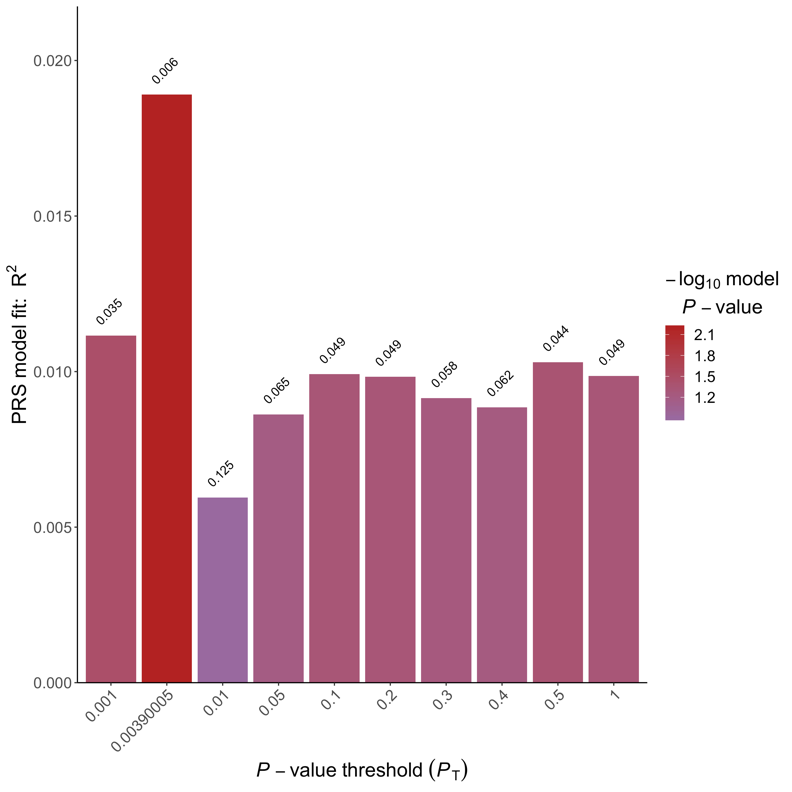


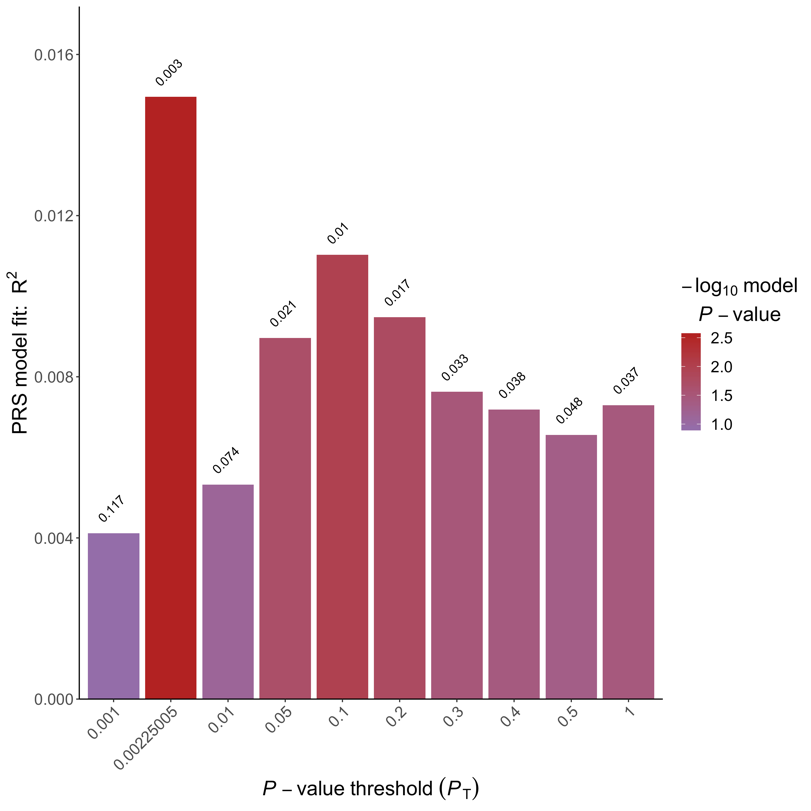


**Supplementary Figure S3.** Polygenic risk score distribution divided into low (quintile 1; green), intermediate (quintile 2-4; blue), and high risk (quintile 5; red) groups for (A) posttraumatic stress and (B) depressive symptoms as derived using the PTSD GWAS.

**A**

**B**

**A**

**B**

**Supplementary Figure S4. Odds ratios for each quintile of the polygenic risk scores for PTS and DS as derived using the PTSD GWAS.** The first quintile is used as a reference. For every other quintile, the odds ratio relative to the first quintile is displayed for (A) posttraumatic stress (PTS) and (B) depressive symptoms (DS). Individuals in the 5th quintile have 2.8 times higher odds of developing PTS and 3.5 times higher odds of developing DS than individuals in the first quintile. The error bars indicate 95% confidence intervals around the odds ratios. Individuals were categorized into three risk groups: low (quintile 1; green), mid (quintiles 2-4; blue), and high (quintile 5; red).

**Supplementary Table S1.** PRS prediction of posttraumatic stress (PTS), depressive symptoms (DS), and musculoskeletal pain (MSP) following motor vehicle collision in participants with accident severity data.

|  | **PTS (n=751)** | | **DS (n=756)** | | **MSP (n=754)** | |
| --- | --- | --- | --- | --- | --- | --- |
| **Variable** | **aOR (95%CI)** | **p-value** | **aOR (95%CI)** | **p-value** | **aOR (95%CI)** | **P -value** |
| PTSD-PRS | 1.43 (1.15-1.80) | 0.00167 | 1.43 (1.10-1.86) | 0.00778 | 0.89 (0.76-1.04) | 0.130442 |
| MDD-PRS | 1.15 (0.95-1.43) | 0.21802 | 1.32 (1.03-1.70) | 0.02764 | 1.14 (0.97-1.33) | 0.106364 |
| Back Pain-PRS | 1.06 (0.87-1.32) | 0.56335 | 1.21 (0.95-1.54) | 0.12433 | 1.23 (1.06-1.44) | 0.007583 |
| Age | 1.01 (0.99-1.02) | 0.38749 | 1.00 (0.98-1.02) | 0.86526 | 1.01 (1.00-1.03) | 0.023192 |
| Sex (male) | 1.06 (0.69-1.65) | 0.80270 | 1.09 (0.66-1.81) | 0.74280 | 1.69 (1.23-2.34) | 0.001494 |
| Moderate accident | 1.28 (0.65-2.66) | 0.48073 | 1.59 (0.77-3.52) | 0.22754 | 0.88 (0.55-1.42) | 0.605711 |
| Severe accident | 1.26 (0.66-2.54) | 0.49005 | 1.11 (0.55-2.41) | 0.78789 | 0.77 (0.77-4.95) | 0.239768 |

aOR=adjusted odds ratio. 95% CI=95% confidence interval. PTSD-PRS, MDD-PRS, and Back Pain-PRS are polygenic risk scores derived from PTSD, MDD, and back pain GWAS summary statistics for PTS, DS, and MSP outcomes. Model was adjusted for enrollment site and top ten principal components, but these variables were omitted from this table for brevity.

**Supplementary Table S2.** Adjusted logistic regression models for polygenic risk derived from PTSD GWAS and protective factor on PTS.

|  | **Neighborhood (n=776)** | | **Social Support (n=774)** | | **Education (n=775)** | |
| --- | --- | --- | --- | --- | --- | --- |
| **Variable** | **aOR (95%CI)** | **p-value** | **aOR (95%CI)** | **p-value** | **aOR (95%CI)** | **p-value** |
| PTSD-PRS for PTS | 1.42 (1.14-1.78) | 0.00164 | 1.41 (1.14-1.77) | 0.00199 | 1.43 (1.15-1.78) | 0.00158 |
| Protective factor | 0.74 (0.48-1.14) | 0.16922 | 0.98 (0.60-1.63) | 0.92756 | 0.46 (0.28-0.75) | 0.00194 |
| Age | 1.00 (0.99-1.02) | 0.56525 | 1.00 (0.99-1.02) | 0.62668 | 1.00 (0.99-1.02) | 0.32688 |
| Sex (male) | 1.03 (0.67-1.60) | 0.89507 | 1.04 (0.68-1.61) | 0.85599 | 1.20 (0.77-1.87) | 0.42631 |

aOR=adjusted odds ratio. 95% CI=95% confidence interval. Models were adjusted for enrollment site and top 10 principal components, but these variables were omitted from this table for brevity.

**Supplementary Table S3.** Adjusted logistic regression models for polygenic risk derived from PTSD GWAS and protective factor on DS.

|  | **Neighborhood (n=781)** | | **Social Support (n=779)** | | **Education (n=780)** | |
| --- | --- | --- | --- | --- | --- | --- |
| **Variable** | **aOR (95%CI)** | **p-value** | **aOR (95%CI)** | **p-value** | **aOR (95%CI)** | **P -value** |
| PTSD-PRS for DS | 1.54 (1.19-1.99) | 0.000935 | 1.54 (1.20-2.00) | 0.000947 | 1.54 (1.20-1.98) | 0.000878 |
| Protective factor | 0.75 (0.46-1.21) | 0.243584 | 0.38 (0.23-0.62) | 8.72 x 10^-5^ | 0.54 (0.31-0.91) | 0.023594 |
| Age | 1.00 (0.98-1.01) | 0.790215 | 1.00 (0.98-1.02) | 0.901517 | 1.00 (0.98-1.02) | 0.979279 |
| Sex (male) | 1.08 (0.67-1.76) | 0.766304 | 1.20 (0.74-1.97) | 0.474083 | 1.22 (0.75-2.02) | 0.419496 |

aOR=adjusted odds ratio. 95% CI=95% confidence interval. Models were adjusted for enrollment site and top 10 principal components, but these variable were omitted from this table for brevity.

**Supplementary Table S4.** Association between PTSD-PRS for PTS and DS and neighborhood socioeconomic status (SEP index), social support (MSPSS score), and educational attainment (highest grade).

| **PRS** | **Environmental Factor** | **Correlation coefficient (r)** | **p-value** |
| --- | --- | --- | --- |
| PTS | Neighborhood SES | -0.002 | 0.953 |
| PTS | Social support score | -0.008 | 0.830 |
| PTS | Highest grade | 0.017 | 0.640 |
| DS | Neighborhood SES | 0.018 | 0.622 |
| DS | Social support score | -0.025 | 0.493 |
| DS | Highest grade | 0.003 | 0.939 |

r = Spearman correlation coefficient
